# Recovery of physical function in lung transplant recipients with sarcopenia

**DOI:** 10.1186/s12890-021-01442-5

**Published:** 2021-04-16

**Authors:** Etsuhiro Nikkuni, Takashi Hirama, Kazuki Hayasaka, Sakiko Kumata, Shinichi Kotan, Yui Watanabe, Hisashi Oishi, Hiromichi Niikawa, Masahiro Kohzuki, Yoshinori Okada

**Affiliations:** 1 Department of Rehabilitation, Tohoku University Hospital, Sendai, Miyagi Japan; 2 Department of Thoracic Surgery, Institute of Development, Aging and Cancer, Tohoku University, Sendai, Miyagi Japan; 3 Division of Organ Transplantation, Tohoku University Hospital, Sendai, Miyagi Japan; 4 Department of Internal Medicine and Rehabilitation Science, Tohoku University Graduate School of Medicine, Sendai, Miyagi Japan

**Keywords:** Lung transplant, Sarcopenia, Erector spine muscle (ESM), Hand-grip, Six-min walk distance (6MWD), Muscle

## Abstract

**Background:**

Lung transplant (LTX) can provide a survival benefit and improve physical function for selected patients with advanced pulmonary disease. Sarcopenia is a systemic muscle-failure that can be found in a variety of life stages and disabilities. In this study, we follow the evolution of each variable defined in sarcopenia and the outcomes in LTX recipients with post-transplant sarcopenia.

**Methods:**

Patients who underwent LTX at Tohoku University Hospital between 2013 and 2018 were consecutively included in the retrospective cohort study, with follow-up to 2019. Sarcopenia was defined by low muscle mass (the cross-sectional area (CSA) of erector spinae muscle (ESM) in thoracic CT with a threshold < 17.24 cm^2^/m^2^) and either low muscle strength (hand-grip with a threshold of < 26 kg in males and of < 18 kg in females) or physical performance (6-min walk distance with a threshold < 46.5% of predicted distance).

**Results:**

Fifty-five recipients were included into the study, of whom 19 patients were defined as sarcopenic and 36 as non-sarcopenic. The muscle mass improved after transplant in both sarcopenic and non-sarcopenic individuals: the median ESM-CSA enlarged from 17.25 cm^2^/m^2^ in 2 months post-LTX to 18.55 cm^2^/m^2^ in 12 months (*p* < 0.001) and 17.63 cm^2^/m^2^ in 36 months (*p* < 0.001) in non-sarcopenic individuals, while in sarcopenic patients it improved from 13.36 cm^2^/m^2^ in 2 months to 16.31 cm^2^/m^2^ in 12 months (*p* < 0.005) and 18.01 cm^2^/m^2^ in 36 months (*p* < 0.001). The muscle mass in sarcopenia substantially recovered to close to non-sarcopenic conditions within 36-months (*p* < 0.001 in 2 months and *p* = 0.951 in 36 months). Accordingly, muscle strength and physical performance in both groups improved over time. No difference in survival was seen in both groups (Log-rank *p* = 0.096), and sarcopenia was not associated with an overall hazard of death (*p* = 0.147). There was no difference in the cumulative incidence of chronic lung allograft dysfunction between patients with or without sarcopenia (Log-rank *p* = 0.529).

**Conclusions:**

Even patients with post-transplant sarcopenia have a chance to recover physical function to levels close to those without sarcopenia several years post LTX.

**Supplementary Information:**

The online version contains supplementary material available at 10.1186/s12890-021-01442-5.

## Background

Lung transplant (LTX) can provide a survival benefit and improve physical and functional ability for selected patients with advanced pulmonary disease. Physical strength is one of the essential capacities to live as a human being, yet its evaluation with clinical tests is technically difficult as measuring muscle quantity and quality with high accuracy is not well standardized [1]. Sarcopenia is a systemic muscle-failure and is, although previously considered to be primarily associated with age-associated muscle disease in older people, currently found in a variety of life stages [2] and disabilities[3–5]. As documented in guidelines [6–8], measurement of muscle quantity, muscle strength and physical performance is recommended for the diagnosis of sarcopenia.

Although LTX is becoming increasingly common, Japan has a unique profile owing to a severe donor-shortage with 0.99 donations per million [9]: a strict age limit has been set up for listing (younger than 55 years old for a double LTX and 60 for a single), the number of single LTX outnumbers that of the double, and the mean waiting time is over 900 days [10, 11]. Therefore, LTX recipients in Japan are relatively young but become physically weakened during long waiting periods. Given those facts, it is conceivable that many LTX recipients, although this has not been evaluated, became post-operatively sarcopenic due to the progression of pulmonary disease during the long waiting time and/or post-transplant complications.

A recent systematic review noted that few studies have examined the prevalence of sarcopenia in LTX using the consensus definition and indicated that additional studies to assess sarcopenia with standardization of measurement techniques and incorporation of clinical outcomes were needed [12]. Therefore, the threshold for each variable to define sarcopenia was setup, and the medical chart of LTX recipients were retrospectively reviewed. We aimed to see how each variable related to physical and functional ability changed with each passing year and herein report the outcomes in LTX recipients with post-transplant sarcopenia.

## Methods

### Study design and data collection

Patients who underwent LTX at Tohoku University Hospital (TUH) between January 2013 and December 2018 were consecutively included in the retrospective cohort study, with follow-up extending to December 2019. LTX recipients who were younger than 18 years old or died within a year after transplant were excluded from the study. Baseline data were collected at the time of transplantation, and follow-up data were gathered monthly until hospital discharge, as well as at month 6, and annually post-transplant. All methods were performed in accordance with the Declaration of Helsinki. In light of the retrospective design, the requirement of informed consent was waived and the study protocol was approved by the Ethics Committee Tohoku University Graduate School of Medicine (Institutional Review Board number 2020-1-388). We disclosed information on the implementation of the research and ensured the opportunity for research subjects to refuse participation by posting the information disclosure materials approved by the Ethics Committee on the website of the Graduate School of Medicine, Tohoku University.

### Management of LTX and definition of variables

All recipients received the same immunosuppression protocol in our centre[16], 16, with basiliximab for induction. Immunosuppression was maintained using tacrolimus targeting C0 level of 10–14 ng/ml for the first 6 months, 9–13 ng/ml up to 12 months and 8–10 ng/ml thereafter, mycophenolate at 1500 mg ≥ 50 kg or 1000 mg for < 50 kg as tolerated and prednisolone at 1.0 mg/kg for the first 4 days, tapering gradually to 5 mg. When the patients could not tolerate tacrolimus or mycophenolate, cyclosporine or azathioprine was the alternative, respectively. No recipients received rituximab or rabbit anti-thymocyte globulin in the study period. Duration of ventilation was defined as the first day of extubation from the invasive mechanical ventilation after the surgery. The requirement of continuous renal replacement therapy (CRRT) for post-operative acute kidney injury was previously documented [16]. The presence of primary graft dysfunction at 24, 48 and 72 h after the graft perfusion was evaluated based on ISHLT consensus [18]. Acute allograft rejection, considered when there was an acute drop in lung function without episodes of infection or mechanical complications including pleural effusion, airway stenosis or native lung hyperinflation, was treated with a bolus of methylprednisolone at 500 mg for 3 consecutive days, followed by tapering doses of prednisone back down to 5 mg. Chronic lung allograft dysfunction (CLAD) was defined by a substantial (≥ 20%) and persistent (≥ 3 months) decline in FEV1 from the baseline value beyond the first year post-transplant [19].

### Definition of sarcopenia

**Fig. 1:**
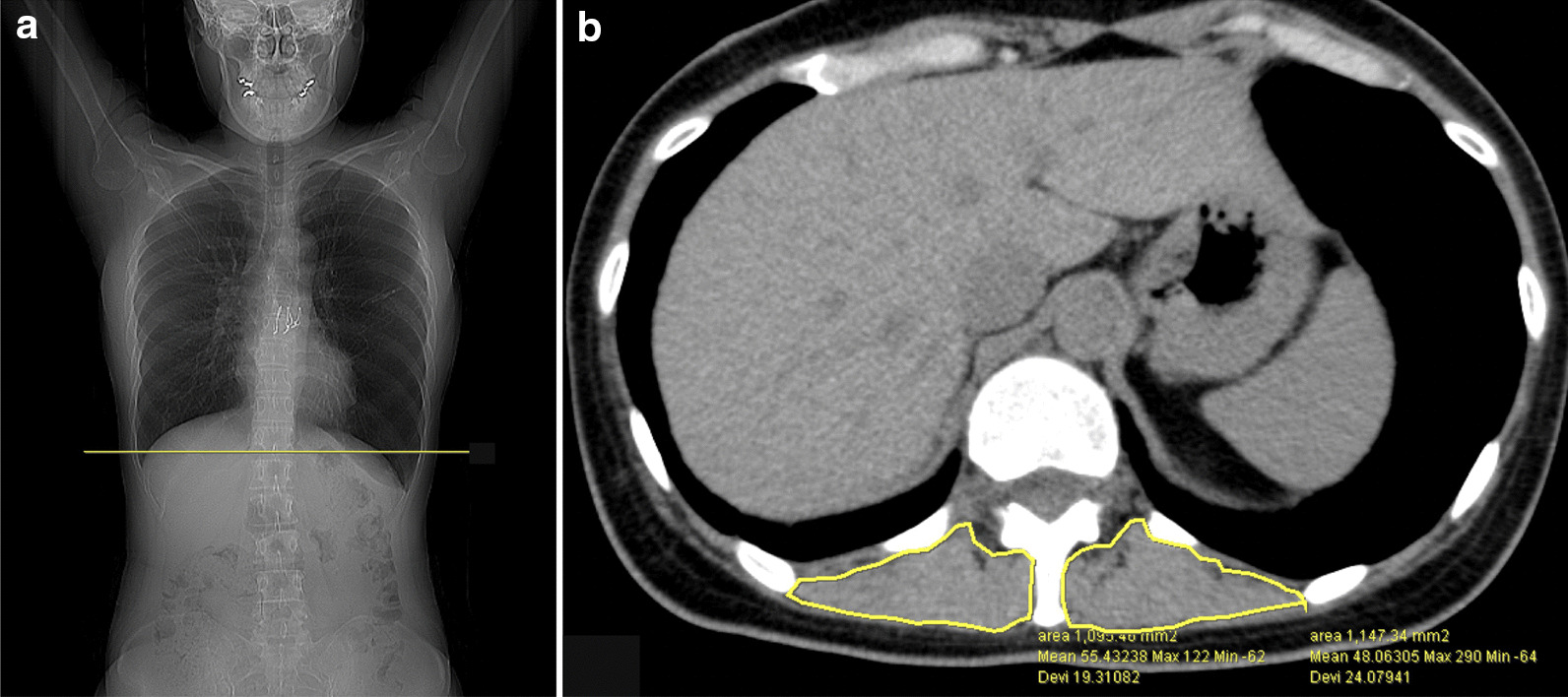
The measurement of the cross-sectional area (CSA) of erector spinae muscle (ESM) in thoracic CT. (**a**) The image in thoracic CT at the lower margin of the 12th thoracic vertebra was viewed with WeVIEW Z-edition (Hitachi, Ltd, Tokyo, Japan). (**b**) The border of ESM-CSA was outlined (shown in yellow lines), and the area was automatically calculated

Post-transplant sarcopenia was assessed at month 2 and 6, and annually after LTX. Sarcopenia was defined by low muscle mass and either low muscle strength or physical performance [6, 7] (Table 1). LTX recipients who did not fulfil the definition of sarcopenia were categorized as non-sarcopenia. The low muscle mass was characterized by the cross-sectional area (CSA) of erector spinae muscle (ESM) in thoracic CT with a threshold < 17.24 cm^2^/m^2^ based on the study from the Japanese population [13]. Images of ESM-CSA were acquired on Toshiba Aquilion ONE scanner at each clinical assessment, analyzed with WeVIEW Z-edition (Hitachi, Ltd, Tokyo, Japan) (Fig. 1). Briefly, the border of ESM-CSA was outlined, and the area (cm2) was then normalized to body surface area (BSA) [13]. Muscle strength was assessed using peak hand-grip force on the dominant hand with a dynamometer (Takei Scientific Instruments Co.,Ltd Nigata, Japan), and the low muscle strength was determined by hand-grip with a threshold of < 26 kg in males and of < 18 kg in females [7]. The trend of hand-grip strength was followed with actual and %predicted values [14]. The low physical performance was determined by 6-min walk distance (6MWD) with a threshold < 46.5% of predicted distance [15].

**Table 1:** The definition of sarcopenia in lung transplant recipients

Variable	Clinical practice	Threshold
[1] Muscle mass	The cross-sectional area of erector spinae muscle/body surface area	< 17.24 cm^2^/m^2^
[2] Muscle strength	Handgrip strength	Men: < 26 kg, Women: < 18 kg
[3] Physical performance	% predicted six-min walk distance	< 46.5%

Sarcopenia was defined by [1] low muscle mass and either [2] low muscle strength or [3] physical performance

### Post-transplant rehabilitation

Physical rehabilitation was started within 24 h post-operatively, aiming early mobilization, secretion clearance, breathing exercises, building upper and lower extremity range of motion and posture improvement. After being discharged from intensive care unit (ICU), reassessing supplemental oxygen requirements, balancing activities and transferring from bed to chair were included in the early in-patient rehabilitation. Lower extremity strength and gait speed were daily assessed in the rehabilitation unit in the late in-patient rehabilitation in order for the recipients to be deemed safe and independent for discharge home. ESM-CSA through thoracic CT, hand-grip strength, 6MWD, lung function and body mass index (BMI) were assessed monthly until hospital discharge, as well as at month 6, and annually post-transplant. After the surgery, LTX recipients remained in the hospital until adequate physical recovery and post-transplant rehabilitation continued to be provided during the hospital stay.

## Data analysis

The variables between sarcopenia and non-sarcopenia were shown in percentage or medians (interquartile range (IQR)) as appropriate, and the difference in baseline data were assessed with chi-square or Fisher’s exact tests for categoric variables and Mann–Whitney U test for continuous variables. Changes in follow-up data with respect to the first fully clinical/physical assessment done in 2 months post-LTX were analyzed by Wilcoxon signed-rank test, and the differences across groups by Mann–Whitney U test. Risk factors associated with post-transplant sarcopenia were assessed using multivariable logistic regression models, and those for mortality were analyzed using a Cox proportional hazards model. The Kaplan–Meier method was used to model time-to-event outcomes, and differences across groups were calculated with the log-rank test. Unadjusted survival analyses were performed to avoid overfitting due to the small sample size. P values of < 0.05 were considered statistically significant. Statistical analyses and graph generation were performed with GraphPad Prism 6.0 (GraphPad Software, Inc., La Jolla, CA), Jamovi (Version 0.9, retrieved from https://www.jamovi.org) and StatPlus:macLE (AnalystSoft; Walnut, California, US).

## Results

### Characteristics of patients with sarcopenia versus non-sarcopenia

**Fig. 2:**
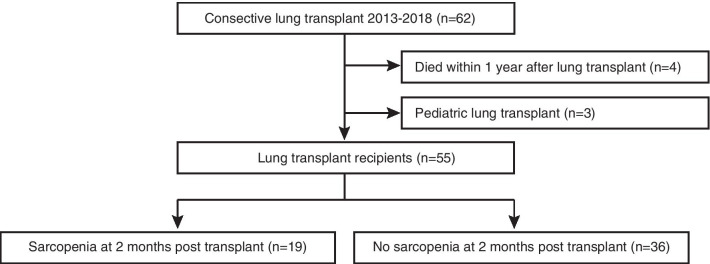
Study flow

Sixty-two patients underwent LTX at TUH between 2013 and 2018, from whom 4 died within a year after transplantation (one died due to invasive aspergillosis, another due to esophageal fistula and the others due to primary graft dysfunction) and 3 pediatric cases were excluded from the analysis. Other 55 LTX recipients were included in the study, of whom the status of whether they were sarcopenia or not was assessed at 2 months post-LTX. Of these, 19 patients were defined as sarcopenic and 36 as non-sarcopenic (Fig. 2, Table 2). Median age at the time of LTX was 44 (IQR 33–50) without difference between sarcopenic and non-sarcopenic individuals (*p* = 0.878). Females accounted for 52.7% (29/55) of all recipients, with female dominance in sarcopenia (63.2%, 12/19) relative to non-sarcopenia (47.2%, 17/36) albeit without significance (*p* = 0.260). Transplant procedures including 49.1% of single (27/55), 49.1% of double (27/55) and 1.8% of living-donor (1/55) were done almost equally in both groups (*p* = 0.528). There was no statistic difference in LTX between groups (*p* = 0.550), with pulmonary vascular disease at 18.2% (10/55), restrictive at 25.5% (14/55), obstructive at 36.4% (20/55), suppurative at 3.6% (2/55) and others at 5.4% (3/55). Despite sarcopenia defined by low muscle mass and physical function, pre-transplant body mass index and walk distance were not different at the time of listing (*p* = 0.815 and 0.524, respectively). Median waiting time was similar with 26 months (IQR 16–68) in sarcopenia and 26 (IQR 17–35) in non-sarcopenia. No LTX recipients were bridged on extracorporeal membrane oxygenation (ECMO) or mechanical ventilation (MV). Moreover, no significant difference was found in donor age (*p* = 0.411) and ischemic time (*p* = 0.502) between both groups. Post-operative condition and complications were reviewed, showing that ICU stay was longer in sarcopenia (29 days (IQR 16–68)) than that of non-sarcopenia (15 days (IQR 7–31), *p* = 0.024). Length of MV and hospital stay was numerically longer in sarcopenia but not statistically (*p* = 0.052 and 0.060, respectively). It was notable that no risk factors associated with post-transplant sarcopenia at 2 months were identified among variables including age, gender, LTX procedure, ICU stay and the length of hospitalization (Table 3).

**Table 2:** Patients’ characteristics with sarcopenia (n = 19) and non-sarcopenia (n = 36)

	Total (n = 55)	Sarcopenia (n = 19)	Non-sarcopenia (n = 36)	*P*-value
*Patients' characteristics*				
Age at LTX, median (IQR)	44 (33–50)	45 (34–50)	44 (33–50)	0.878
Female, n (%)	29 (52.7%)	12 (63.2%)	17 (47.2%)	0.260
LTX procedure, n (%)				0.529
Single	27 (49.1%)	8 (42.1%)	19 (52.8%)	
Double	27 (49.1%)	11 (57.9%)	17 (47.2%)	
Living-donor	1 (1.8%)	1 (5.3%)	0 (0.0%)	
LTX indication, n (%)				0.550
Pulmonary Vascular Disease	10 (18.2%)	5 (26.3%)	5 (13.9%)	
Restrictive Lung Disease	14 (25.5%)	4 (21.1%)	10 (27.8%)	
Obstructive Lung Disease	20 (36.4%)	8 (42.1%)	12 (33.3%)	
Suppurative Lung Disease	8 (14.5%)	1 (5.3%)	7 (19.4%)	
Others	3 (5.4%)	1 (5.3%)	2 (5.6%)	
*Pre- and intra-operative condition*				
6MWD (m), median (IQR)	318 (216–373)	316 (221–387)	318 (204–369)	0.524
Body-mass index (kg/m^2^), median (IQR)	18.0 (17.0–22.0)	18.0 (17.0–24.0)	18.5 (16.3–21.0)	0.815
On supplemental oxygen, n (%)	49 (89.1%)	17 (89.5%)	32 (88.9%)	0.999
Diabetes, n (%)	4 (7.3%)	3 (15.8%)	1 (2.8%)	0.077
Connective tissue disease, n (%)	9 (16.4%)	3 (15.8%)	6 (16.7%)	0.933
Chronic kidney disease, n (%)	2 (3.6%)	2 (10.5%)	0 (0.0%)	0.047
Waiting time (month), median (IQR)	26 (18–36)	26 (19–37)	26 (17–35)	0.559
Donor age, median (IQR)	43 (31–47)	43 (26–49)	42.5 (31.5–51)	0.411
Ischemic time (min)	535 (452–697)	483 (403–717)	555 (456–694)	0.323
CMV mismatch (D + /R-), n (%)	10 (18.2%)	5 (26.3%)	5 (13.9%)	0.288
*Post-operative condition and complications*				
Primary graft dysfunction, n (%)	45 (81.8%)	17 (89.5%)	28 (77.8%)	0.465
Requirement of tracheostomy, n (%)	28 (50.9%)	13 (68.4%)	15 (42.7%)	0.089
Continuous renal replacement therapy, n (%)	9 (16.4%)	5 (26.3%)	4 (11.1%)	0.249
Invasive mechanical ventilation (day), median (IQR)	12 (4–29)	18 (5–60)	7 (4–21)	0.052
ICU stay (day), median (IQR)	19 (9–39)	29 (16–68)	15 (7–31)	**0.024**
Hospital stay (day), median (IQR)	85 (64–114)	98 (64–170)	81 (62–95)	0.060
Acute allograft rejection, n (%)	9 (16.4%)	3 (15.8%)	6 (16.7%)	0.999
ESM-CSA ( cm^2^/m^2^), median (IQR)	15.7 (14.0–17.9)	13.6 (12.6–14.9)	17.3 (14.8–18.8)	** < .0001**
%predicted hand-grip strength, median (IQR)	54.0 (43.5–62.0)	39.0 (28.0–49.0)	58.0 (52.0–67.0)	** < .0001**
Hand-grip strength (kg), median (IQR)	20.0 (15.0–28.0)	14.0 (11.8–17.3)	24.0 (20.0–29.0)	** < .0001**
%predicted 6MWD, median (IQR)	65.0 (48.0–79.0)	39.0 (31.0–64.0)	74.5 (62.0–81.8)	** < .0001**
6MWD (m), median (IQR)	447 (309–483)	309 (195–399)	476 (426–519)	** < .0001**
Body-mass index (kg/m^2^), median (IQR)	17.0 (15.0–21.0)	17.0 (15.0–22.0)	17.0 (16.0–20.0)	0.891
FEV1 (L), median (IQR)	1.56 (1.30–1.95)	1.36 (1.13–1.56)	1.78 (1.37–2.02)	**0.013**
Follow-up duration in months, median (IQR)	48 (26–70)	43 (22–76)	49 (27–67)	0.571

Bold values of *p* < 0.05 were considered statistically significant

LTX, lung transplant; IQR, interquartile range; 6MWD, 6-min walk distance; CMV, cytomegalovirus; D, donor; R; recipient, ICU, intensive care unit; FEV1, forced expiratory volume in one second

**Table 3:** Risk factors associated with post-transplant sarcopenia

	OR	95% CI	*p* value
Age at LTX	1.00	0.94–1.06	0.996
Gender, female	2.12	0.58–7.60	0.251
LTX procedure, double	0.79	0.17–3.65	0.765
ICU stay	1.02	0.98–1.06	0.398
Hospital stay	1.01	0.99–1.02	0.235

LTX, lung transplant; ICU, intensive care unit; OR, odds ratio and CI, confidence interval. Living-donor LTX (n = 1) was included double LTX

### A trend in ESM-CSA in patients after lung transplant

**Fig. 3:**
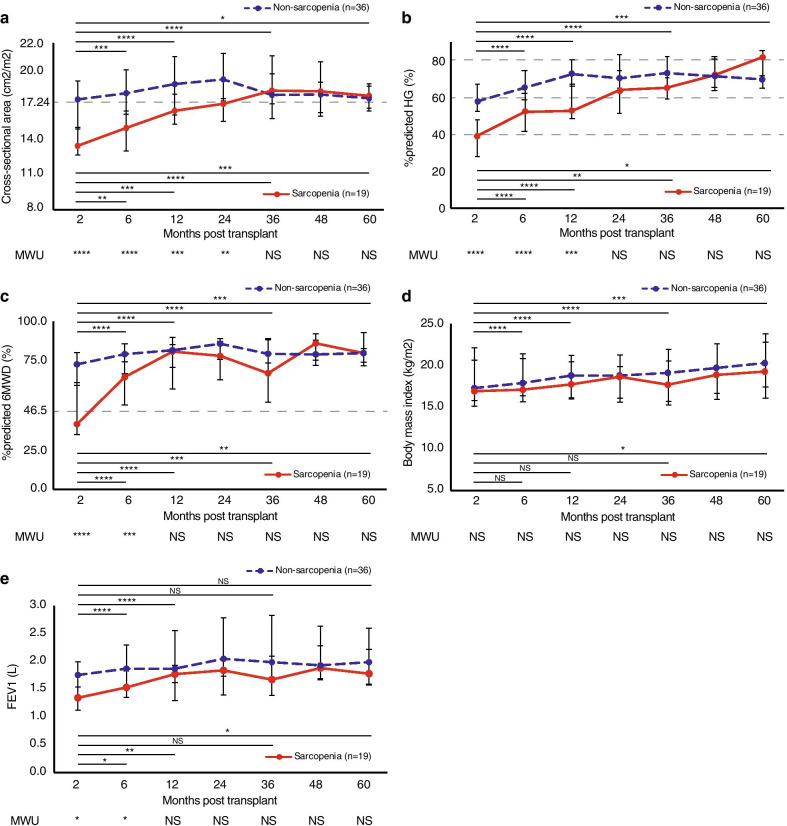
Trend of variables after lung transplant in patients with and without sarcopenia. **a** The trend in the cross-sectional area of erector spinae muscle normalized to body surface area (cm^2^/m^2^), **b** % predicted hand-grip (HG) strength, **c** % predicted 6-min walk distance (6MWD), **d** body-mass index (BMI)(kg/m2) and **e** forced expiratory volume in the first second (FEV1)(L) were shown by months after transplant. The change at each annual assessment was compared to the first full assessment normally done in 2- or 3- months after transplant. Sarcopenia was shown in red solid line (n = 19) and non-sarcopenia in blue dashed line (n = 36). The difference between sarcopenia and non-sarcopenia was calculated with Mann–Whitney U test (MWU), described below the line graph. NS, not significantly different, *p < 0.05, **p < 0.01, ***p < 0.005 and ****p < 0.001

The status of whether they were considered post-transplant sarcopenia or not was assessed in all recipients at month 2, and variables that can be associated with sarcopenia were compared at month 6 and annually after LTX. Muscle mass plays an important role in appraising sarcopenia and the trend in ESM-CSA through thoracic CT was shown in Fig. 3a, illustrating that ESM-CSA improved over time in non-sarcopenic recipients for up to 24 months after transplantation (e.g. median ESM-CSA of 17.78 cm^2^/m^2^ in 6 months (*p* < 0.005), 18.55 cm^2^/m^2^ in 12 months (*p* < 0.001) and 17.63 cm^2^/m^2^ in 36 months (*p* < 0.001), compared to 17.25 cm^2^/m^2^ in 2 months after LTX). Likewise, the area of thoracic muscle drastically enlarged after LTX in sarcopenic patients from median ESM-CSA of 13.36 cm^2^/m^2^ in 2 months to 14.86 cm/m^2^ in 6 months (*p* < 0.01), 16.31 cm^2^/m^2^ in 12 months (*p* < 0.005) and 18.01 cm^2^/m^2^ in 36 months (*p* < 0.001). Although ESM-CSA in non-sarcopenic conditions was significantly more massive than that of sarcopenic individuals at 2 months after transplantation (median of 17.25 cm^2^/m^2^ in non-sarcopenia vs 13.36 cm^2^/m^2^ in sarcopenia, *p* < 0.001), the difference became less significant with each passing year and was almost negligible 36 months after LTX (median of 17.63 cm^2^/m^2^ in non-sarcopenia vs 18.01 cm^2^/m^2^ in sarcopenia, *p* = 0.951). This indicated that even LTX recipients who were sarcopenic after the transplant procedure would be capable of rebuilding muscle volume years after transplantation.

### A trend in factors associated with sarcopenia after lung transplant

The trends in the muscle strength demonstrated by predicted hand-grip and physical performance with predicted 6MWD were shown in Fig. 3b, c, respectively. The actual number of median hand-grip strength (kg) and 6MWD (m) was illustrated (Additional file 1: Figure 1). The hand-grip strength displayed an overall upward trend after LTX in both sarcopenic and non-sarcopenic, and the gap of the muscle strength between the groups became less significant at 24 months after transplant. Meanwhile, the physical performance in non-sarcopenia stably improved over the follow-up period and that of sarcopenic individuals has shown a significant recovery. Although the percentage of predicted walk distance in non-sarcopenic excelled that of sarcopenic individuals within a year after LTX (*p* < 0.005), the physical performance was comparable between groups at 12- months and thereafter. The trend in BMI was also reviewed with duration (Fig. 3d), revealing that the BMI has significantly grown in non-sarcopenia (*p* < 0.05 at each year point) but not in sarcopenia. There has been no significant difference in BMI between both groups at any time points post-transplant. The trend in lung function was then followed (Fig. 3e); lung function in LTX recipients diagnosed with non-sarcopenia and sarcopenia substantially improved at 6- and 12- month (*p* < 0.05) but not thereafter. The value in FEV1 in non-sarcopenia virtually outnumbered that of sarcopenia at 2- and 6- month (*p* < 0.01 and < 0.05, respectively) but not significantly afterward.

### The transition of sarcopenic status in lung transplant recipients

**Fig. 4:**
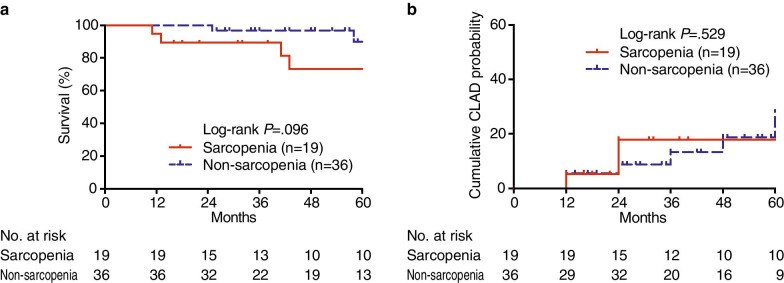
Kaplan–Meier analysis in lung transplant recipients with and without sarcopenia. **a** Survival and **b** the cumulative incidence of chronic lung allograft dysfunction (CLAD) were shown by months after transplant. Sarcopenia was shown in red solid line (n = 19) and non-sarcopenia in blue dashed line (n = 36). The number of patients at risk is documented according to time

The sarcopenic status was re-evaluated in 12-month after LTX (Table 4). Nine (47.4%) out of 19 patients with sarcopenia were re-defined as sarcopenia, whereas 10 recipients (52.6%) were categorized as non-sarcopenic. On the other hand, 35 non-sarcopenic patients (97.2%) have remained in a status of non-sarcopenia and 1 recipient (2.8%) became sarcopenic. In a survival analysis with follow-up until December 2019, the sarcopenic status after LTX was not associated with higher mortality rates than non-sarcopenia (Log-rank *p* = 0.096) (Fig. 4a). Risk factors for mortality after LTX were analyzed in a Cox model (Table 5), demonstrating that age was associated with mortality in both univariate (hazard ratio (HR) 1.12, 95% CI 1.02–1.24, *p* = 0.020) and multivariate analysis (HR 1.19, 95% CI 1.03–1.38, *p* = 0.021). Meanwhile, post-transplant sarcopenia, gender, transplant type and the length of ICU stay were not related to mortality. There was no difference in the cumulative incidence of CLAD between patients with or without sarcopenia (Log-rank *p* = 0.529) (Fig. 4b).

**Table 4:** Outcome of patients with sarcopenia one year after lung transplant

2 months after LTX	12 months after LTX	
	Sarcopenia	Non-sarcopenia
Sarcopenia (n = 19)	9 (47.4%)	10 (52.6%)
Non-sarcopenia (n = 36)	1 (2.8%)	35 (97.2%)

LTX, lung transplant

**Table 5:** Hazard ratio for mortality from univariate and multivariate Cox model

	HR	Univariate	*p* value	HR	Multivariate	*p* value
		95% CI			95% CI	
Sarcopenia	3.83	0.70–20.93	0.122	5.36	0.55–51.93	0.147
Age at LTX	1.12	1.02–1.24	0.020	1.19	1.03–1.38	0.021
Gender, female	0.47	0.09–2.55	0.380	0.30	0.04–2.22	0.236
LTX procedure, double	1.51	0.27–8.26	0.638	3.08	0.23–41.9	0.398
ICU stay	1.02	0.99–1.05	0.139	1.01	0.97–1.05	0.597

LTX, lung transplant; ICU, intensive care unit; HR, hazard ratio and CI confidence interval. Living-donor LTX (n = 1) was included double LTX

## Discussion

The muscle mass and strength related to sarcopenia were reviewed in LTX recipients and followed to see outcomes in a transplant centre in Japan. The ESM-CSA represents the muscle content in the thorax and is a vital factor to assess functional strength from the standpoint of sarcopenia. Several variables were used to define sarcopenia in LTX recipients [12]. Although the muscle amount based on the CSA of target muscles through thoracic CT was reported to be associated with the hospital stay or survival after LTX [3, 20–22], these studies examined sarcopenia only by the target muscles mass, not including muscle strength and physical performance. We herein demonstrated that the ESM-CSA steadily improved after transplant in both sarcopenic and non-sarcopenia, and the muscle size in sarcopenia substantially recovered close to that in non-sarcopenic individuals by 36-months. Accordingly, muscle strength and physical performance in both groups improved over time. Importantly, half of the patients with sarcopenia could deviate from such fragile status and be re-defined as non-sarcopenic in 12-months. Furthermore, no difference in survival and the cumulative incidence of CLAD was seen in sarcopenic vs non-sarcopenic patients. With those features in mind, post-transplant sarcopenia did not affect survival and graft failure and, more importantly, even physically and functionally frail patients have a chance to recover their condition close up to those in the non-sarcopenic years after LTX.

Six-minute walking test is a favorable scheme to measure physical functionality for patients with pulmonary disorder and oxygen requirement on exertion [23]. Despite its common use in clinical practice and the fact that pre-transplant long-walk distance was associated with post-transplant survival advantage [24], there are not many studies that evaluate sarcopenia using 6MWD. Therefore, the appropriate threshold of % predicted distance is not clearly studied among lung transplant recipients. With our data, LTX recipients were further categorized as infirmness (n = 18), defined with low muscle mass (ESM-CSA/BSA < 17.24 cm^2^/m^2^) and low muscle strength (Hand-Grip < 26 kg in male and < 18 kg in female) and robustness (n = 9), defined with ESM-CSA/BSA ≥ 17.24 cm^2^/m^2^ and Hand-Grip ≥ 26 kg in male and ≥ 18 kg in female (Additional file 2: Figure 2). Based on that, the threshold at 72% predicted distance was tentatively setup with the upper margin of the cluster of infirmness group that contained the majority of the cases. Using the threshold, 33% (3/9) of the recipients classified as robustness could have poor physical performance, with which we should conduct a prospective study if such threshold would be appropriate to see the physical performance in LTX recipients. On the other hand, 6MWD in both sarcopenic and non-sarcopenic individuals could not reach 100% predicted distance, even several years after LTX. The task of how best to provide the appropriate physiotherapy in the late phase post-transplant should be addressed in the next study.

The study outcomes reminded us of conducting an additional analysis comparing post-transplant sarcopenia with pre-transplant condition. It is, however, not feasible to assess pre-transplant sarcopenia with the participants in the current study due to the transplant allocation system in Japan. As documented above, the mean waiting time is currently over 900 days in the country [11]. Meanwhile, Japan Organ Transplant Network (JOTN) allows LTX candidates to change the waiting status from active to inactive, according to the candidates’ requests. Under the system, some of the participants have been inactive for a while after listing, and the median waiting time is further prolonged. The median waiting time among the participants in the study (n = 55) was 26 months (IQR 19–34). Thus, pre-transplant data available shown in Table 2 were not sufficient and obtaining some variables relating to sarcopenia was not possible when the recipients were listed to JOTN (over 10 years ago in some recipients). We instead gathered information at 2 months after LTX when all recipients were fully assessed, which was compared with variables gathered at 6 months and annually thereafter. In light of the study design, the sarcopenic condition at 2 months may be influenced by not only advanced respiratory disease but also pre-transplant comorbidities, the transplant surgery, and/or post-transplant complications. In fact, ICU stay was significantly longer in the sarcopenic group (*p* = 0.024), and a trend towards prolonged MV and hospitalization was also seen (*p* = 0.052 and 0.060, respectively). Thus, it was uncertain whether pre-transplant condition, post-transplant complications or both led to post-transplant sarcopenia. To see possible associations of clinically important factors (age, gender and transplant type) and length of hospitalization and ICU stay with post-transplant sarcopenia, multivariable analysis was performed, which demonstrated that these variables did not contribute to the development of sarcopenic status post-operatively (Table 3). However, additional variables such as the disease progression, pre-transplant comorbidities and other post-operative condition were unable to be included due to the small sample size, and thus further evaluation with a large number would be needed in future studies. Despite those factors contributing a drop in physical strength after transplant, most of recipients with sarcopenia could recover their functionality and half could be non-sarcopenic by 12 months after LTX.

There are several limitations that warrant discussion. First, given the single center study, the number of patients is still not sufficient. The survival was not statistically significant between post-transplant sarcopenia and non-sarcopenia, but seemed numerically different, which should be further assessed in a larger number of participants. Second, a multicenter study in the whole of Japan would be of great interest to see the fraction of pre- and post-transplant sarcopenia and its clinical characteristics in the larger Japanese population. It can provide more suitable threshold of each variable for sarcopenia among lung transplant recipients. Third, LTX recipients who passed away within 1 year after transplant were excluded as their post-LTX muscle strength and physical function could not be assessed. This may be a selection bias as very sick recipients who were unable to survive over 12 months were not considered for the analysis. They were already critically ill peri-operatively, and thus highly likely to be sarcopenic, due to pre-LTX condition and/or peri-transplant complications including major thoracic surgery or strong immunosuppressants.

## Conclusions

In summary, the threshold of the variables to define sarcopenia was setup and patients with post-transplant sarcopenia were retrospectively followed. All variables including the muscle mass, the muscle strength and the physical performance steadily improved over time after transplant. The muscle content of the thorax in sarcopenic patients substantially recovered to close to that of non-sarcopenic individuals within 36-months, and no difference in survival and the cumulative incidence of CLAD was seen between the groups. Although several limitations should be considered, our study suggests that even patients with sarcopenia have a chance to recover to physical and functional conditions close to those in non-sarcopenic individuals after LTX.

## Supplementary Information

**Additional file 1.** The trend in the hand-grip strength (kg) and 6-minute walk distance (m) shown by months after transplant.

**Additional file 2.** An attempt to setup the threshold of the physical performance based on %predicted 6-minute walk test.

## Data Availability

The datasets used and/or analysed during the current study are available from the corresponding author on reasonable request.

## Abbreviations

BMI: Body mass index
BSA: Body surface area
CLAD: Chronic lung allograft dysfunction
CT: Computed tomography
ESM: Erector spine muscle
FEV1: Forced expiratory volume in the first second
JOTN: Japan organ transplant network
LTX: Lung transplant
6MWD: Six-min walk distance
