# Supplementary material for: Recovery of physical function in lung transplant recipients with sarcopenia
Source: BMC Pulm Med. 2021 Apr 16;21:124. doi: 10.1186/s12890-021-01442-5 (PMC8052749; doi:10.1186/s12890-021-01442-5)
Supplement: Supplementary file 1 — Additional file 1. The trend in the hand-grip strength (kg) and 6-minute walk distance (m) shown by months after transplant. [file 12890_2021_1442_MOESM1_ESM.pdf]

Title

Recovery of Physical Function in Lung Transplant Recipients with Sarcopenia

Authors

Etsuhiro Nikkuni[1], Takashi Hirama[2, 3] ¶, Kazuki Hayasaka[2], Sakiko Kumata[2], Shinichi Kotan[1], Yui Watanabe[2], Hisashi Oishi[2], Hiromichi Niikawa[2], Masahiro Kohzuki[4], Yoshinori Okada[2, 3]

¶ corresponding author

Affiliation

1. Department of Rehabilitation, Tohoku University Hospital, Sendai, Miyagi, Japan
2. Department of Thoracic Surgery, Institute of Development, Aging and Cancer, Tohoku University, Sendai, Miyagi, Japan
3. Division of Organ Transplantation, Tohoku University Hospital, Sendai, Miyagi, Japan
4. Department of Internal Medicine & Rehabilitation Science, Tohoku University Graduate School of Medicine, Sendai, Miyagi, Japan

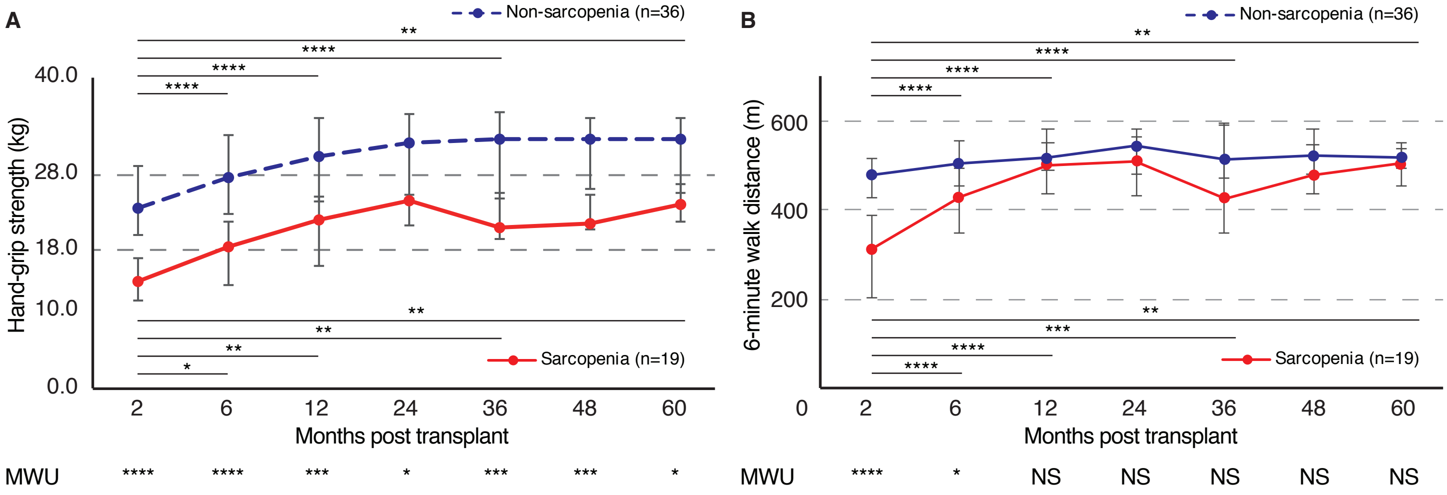

Supplemental figure 1. The trend in the hand-grip strength (kg) and 6-minute walk distance (m) shown by months after transplant

The change at each annual assessment was compared to the first full assessment normally done in 2-month after transplant. Sarcopenia was shown in red solid line (n=19) and non-sarcopenia in blue dashed line (n=36). The difference between sarcopenia and non-sarcopenia was calculated with Mann-Whitney U test (MWU), described below the line graph. NS = not significantly different, *$p < 0.05$, **$p < 0.01$, ***$p < 0.005$ and ****$p < 0.001$.

There has been a persistent difference in the muscle strength throughout the study period between sarcopenia and non-sarcopenia (supplemental figure 1A). This may be due to a gender difference as the threshold was setup independently in men and women. Given female dominance (63.2%, 12/19) in sarcopenia and less (47.2%, 17/36) in non-sarcopenia, better muscle strength in non-sarcopenia could be related to less female dominance in the group. In fact, the difference became less significant with each passing year and was almost negligible 24 months after transplant in the %predicted hand-grip strength (figure 3B).
