# Supplementary material for: Recovery of physical function in lung transplant recipients with sarcopenia
Source: BMC Pulm Med. 2021 Apr 16;21:124. doi: 10.1186/s12890-021-01442-5 (PMC8052749; doi:10.1186/s12890-021-01442-5)
Supplement: Supplementary file 2 — Additional file 2. An attempt to setup the threshold of the physical performance based on %predicted 6-minute walk test. [file 12890_2021_1442_MOESM2_ESM.pdf]

Title

Recovery of Physical Function in Lung Transplant Recipients with Sarcopenia

Authors

Etsuhiro Nikkuni[1], Takashi Hirama[2, 3] ¶, Kazuki Hayasaka[2], Sakiko Kumata[2], Shinichi Kotan[1], Yui Watanabe[2], Hisashi Oishi[2], Hiromichi Niikawa[2], Masahiro Kohzuki[4], Yoshinori Okada[2, 3]

¶ corresponding author

Affiliation

1. Department of Rehabilitation, Tohoku University Hospital, Sendai, Miyagi, Japan
2. Department of Thoracic Surgery, Institute of Development, Aging and Cancer, Tohoku University, Sendai, Miyagi, Japan
3. Division of Organ Transplantation, Tohoku University Hospital, Sendai, Miyagi, Japan
4. Department of Internal Medicine & Rehabilitation Science, Tohoku University Graduate School of Medicine, Sendai, Miyagi, Japan

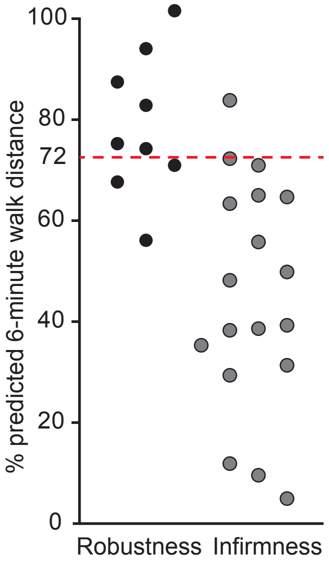

Supplemental figure 2. An attempt to setup the threshold of the physical performance based on %predicted 6-minute walk test

Infirmness in gray circle (n=18) was defined with low muscle mass (by the cross-sectional area (CSA) of erector spinae muscle (ESM)/body surface area (BSA) < 17.24 cm2/m2) and low muscle strength (Hand-Grip <26 kg in male and <18 kg in female), whereas robustness in black circle (n=9) with ESM-CSA/BSA ≥17.24 cm2/m2 and Hand-Grip ≥26 kg in male and ≥18 kg in female. Data were those at months 2 post-transplant.
